# Scientific Items

**Published:** 1880-11-20

**Authors:** 


### SCIENTIFIC ITEMS.

**Science and Spiritualism.**—In his forthcoming work, "The Scientific Basis of Spiritualism," Mr. Epes Sargent takes the ground that natural science is concerned only with the knowledge of realities, that is, of sense-perceptions which can be not only historically but also directly imparted to us, and rationally proved; that, so far as this view is adhered to, Spiritualism is now a science. But he rules out all unconfirmed "trance utterances," or supposed spirit communications, as not pertaining to his "basis," or even coming within the scope of scientific recognition, except so far as they may give clear proof of supersensual power and intelligence.

He selects certain established and daily demonstrable phenomena, about which there is no dispute among those who have investigated the subject scientifically, and makes these the ground for his inductions, as well as the warrant for assuming that other phenomena, equally well attested, but not so perfect and unequivocal in their conditions, are analogically confirmed. He maintains that there are certain preterhuman facts as absolutely proved as any facts in other sciences are proved, and that they are veritable facts of science.

The pretensions of certain so-called "exposers" that they can produce such phenomena as direct writing and clairvoyance by trick, and in the same way that they are medially produced, Mr. Sargent dismisses as being either an ignorant boast or an intentional deception. The facts of the book are those which he had confirmed by forty years of close attention to this subject of Spiritualism, or to the cognate phenomena of mesmerism and somnambulism.

**Self-Winding Clocks.**—A clockmaker of Copenhagen, named Louis Soendenberg, who for some time past has had charge of the city's electric time-keepers, has just invented an ingenious appliance which obviates the necessity of winding up the regulator, from which the clocks in question "take their time."

By a mechanical contrivance which periodically cuts off the stream of electric fluid emanating from the battery, and brings an electric magnet to bear upon the relaxed main-spring in such a way as to renew its tension instantaneously, perpetual motion is practically imparted to the works of the regulator—that is to say, as long as the batteries connected with it are kept properly supplled with acids.

The discoverer of this important improvement has satisfied himself, by six months' successful experiments in his own workshops, that his system works faultlessly, and has applied for permission to adopt it to the electric clocks set up by the municipality in different parts of the Danish capital.

Electricity, under Mr. Soendenberg's compulsion, is destined not only to make the Copenhagen clocks go, but to wind them up, with never-ending recurrence, until the crack of doom.

PAPER stoves are the latest development of German ingenuity.

**The Structure of Spermatozoa.**—The Lancet, July 24, 1880, informs us that in the current number of the Quarterly Journal of Microscopical Science is a short paper by Dr. Heneage Gibbes, in which he states that he has found the spiral filament discovered by him in the spermatozoa of several species of animals, as the rat, mouse, axolotl, pigeon, fowl, snail and leech. In the examination of different specimens of human spermatozoa, he has noticed a variation in the length of the tails, and in one specimen he found a number of heads with no corresponding tails. He throws out the suggestion that these variations may have some important bearing. It is quite possible that tailless spermatozoa may not be able to fertilize the ovum, while the greater the length of the tail, the greater their locomotion and fertilizing power may be.

Dr. O. S. Jensen, of Bergen, has found the spiral filament in the semen of horses; and Professor Fleming, of Kiel, has also confirmed Dr. Gibbes's observations, both as to the existenee of this filament, with its mesentery, and the different reaction to staining fluids of the head and middle part of spermatozoa.—*Med. and Surgical Rep.*

**Prof. Jager's Theory of Man.**—Prof. Jager regards man as a three-fold entity, made up of *body*, of physical substance; *spirit*, or that which is absolutely immaterial and transcendant; and *soul*, or the connecting link between the body and spirit.

The soul is the seat of the will, the passions and the emotions; and it may be isolated by experiment. This is the ancient Sankhya doctrine of the Hindu philosopher, Kapila, afterward reproduced in Egypt, Ionia and Greece, and in the *New Testament*. The spirit, or *purusha*, is divine, and unites as in a womb with the material substance, becoming thus invested with the *linga-sharira*, spiritual form, or psychical entity. Prof. Jager carries his hypothesis to a similar focus with Swedenborg. The soul, or its aura, can be perceived by the sense of smell. The phenomena of sympathy and antipathy between individuals are to be attributed to other constituents of their auras or emanations. The want of harmony in their specific auras, he maintains, is the cause of the social chasm existing between Jews and Christians, Aryans, Negroes, etc.

**Atoms.**—M. COLLADON believes that the fall of hail and rain has an action analogous to that of the *trompe*, and induces a downvard current of air.

A NEW island has been produced in the Azores from terrestrial disturbance in an adjacent island.

SCENIC effects, such as the rising sun, rainbows, etc., are now produced by means of the electric light.

AN INSECT called neen, of the *coccus* species, has been discovered in Yucatan, Central America, which produces a species of india-rubber.

THE Vesuvius railway may soon be tested rather severely; ominous indications are becoming visible of increased activity in the volcano.

THE French Senate have voted $208,000 for a further survey of the Sahara railway route.

THERE are now 171 Scotch blast furnaces in operation, against 90 a year since.—*Journ. of Chemistry.*

